# Supplementary material for: Symmetry-breaking dynamics in a tautomeric 3D covalent organic framework
Source: Nat Commun. 2023 Jul 14;14:4215. doi: 10.1038/s41467-023-39998-x (PMC10349083; doi:10.1038/s41467-023-39998-x)
Supplement: Supplementary file 1 — Supplementary Information [file 41467_2023_39998_MOESM1_ESM.pdf]

Supplementary Information (42 pages)

Symmetry-breaking dynamics in a tautomeric 3D covalent organic framework

Yangyang Xu,[1,‖] Tu Sun,[1,2,‖] Tengwu Zeng,[1,‖] Xiangyu Zhang,[1] Xuan Yao,[1] Shan Liu,[1] Zhaolin Shi,[1] Wen Wen,[3] Yingbo Zhao,[1,2] Shan Jiang,[1,2] Yanhang Ma,[1,2] and Yue-Biao Zhang*,[1,2]

[1]*School of Physical Science and Technology, ShanghaiTech University, Shanghai 201210, China.*

[2]*Shanghai Key Laboratory of High-resolution Electron Microscopy, ShanghaiTech University, Shanghai 201210, China.*

[3]*Shanghai Synchrotron Radiation Facility, Shanghai Advanced Research Institute, Chinese Academic of Sciences, Shanghai 201210, China.*

**Corresponding author:* zhangyb@shanghaitech.edu.cn

[‖]*These authors contributed equally.*

Supplementary Table of Contents

Supplementary Section 1. Synthesis and characterisation S3

Supplementary Section 2. 3D electron diffraction analyses S5

Supplementary Section 3. Powder X-ray diffraction analyses S16

Supplementary Section 4. Gas adsorption and *in-situ* PXRD analyses S24

Supplementary Section 5. Diffuse reflectance spectroscopy S28

Supplementary Section 6. Solid-state NMR spectroscopy S32

Supplementary Section 7. Molecular Dynamics Simulation S40

Supplementary References S42

Supplementary Section 1. Synthesis and characterisation

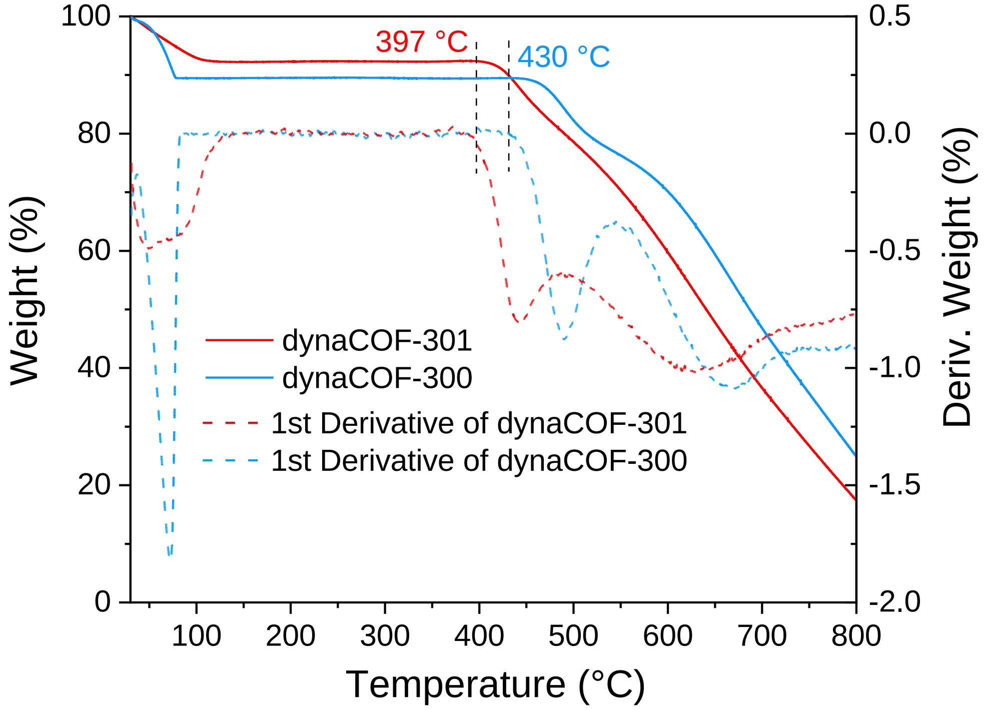

Supplementary Figure 1. Thermal gravimetric and 1st derivative curves of dynadynaCOF-301 (red) and dynaCOF-300 (blue). The thermal stability of dynaCOF-301 is up to 397 °C. Due to moisture uptake from the air, weight losses before 120 °C are attributed to the adsorbed water in pores.

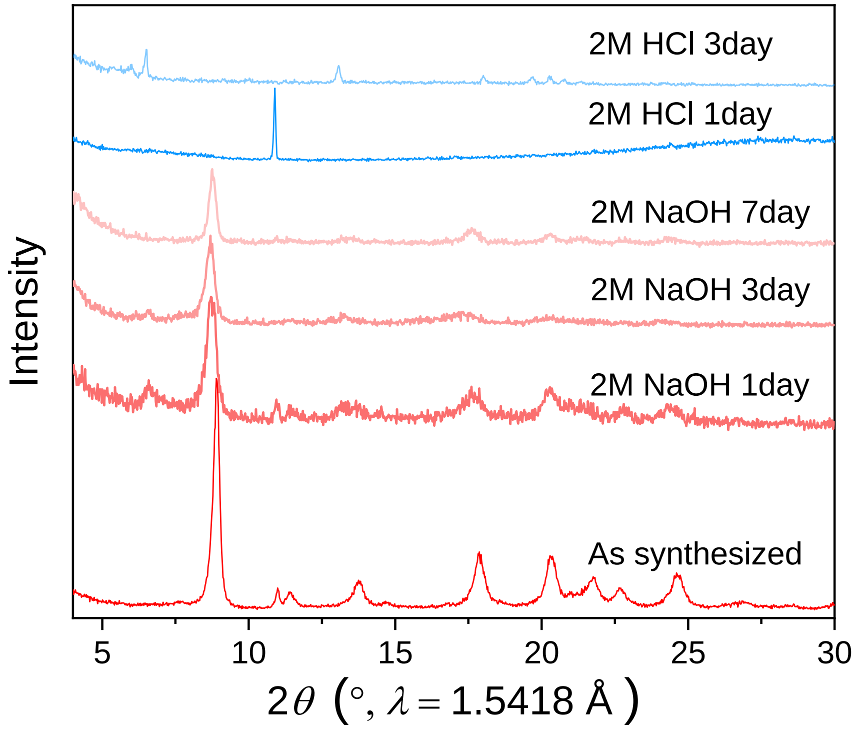

Supplementary Figure 2. PXRD patterns of the dynaCOF-301 immersed in 2 M HCl and 2 M NaOH show that the crystallinity can be partially sustained for at least 7 days in 2 M base (NaOH). Due to the tautomerism of iminol/*cis*-ketoenamine, dynaCOF-301 remains unstable in acidic conditions.

Supplementary Section 2. 3D electron diffraction analyses

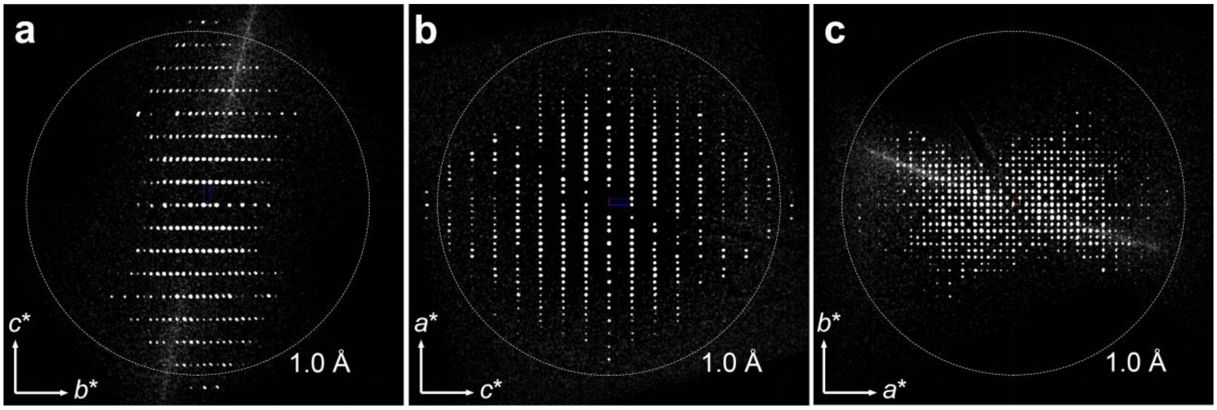

Supplementary Figure 3. Projections of reconstructed three-dimensional reciprocal lattice of dynaCOF-301s along (**a**) [100], (**b**) [010], and (**c**) [001] directions, respectively. The square root of the intensity value of all pixels was used to show the weak diffraction points clearly. The reconstructed reciprocal lattice shows an *I*-centred tetragonal Bravais lattice. And the resolution of the dataset is up to 1.0 Å.

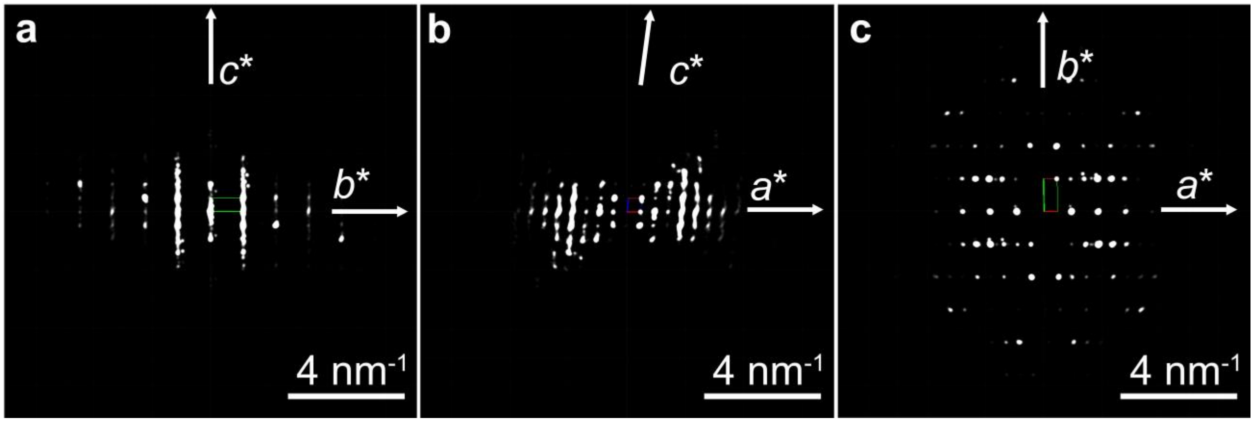

Supplementary Figure 4. The projection of reconstructed three-dimensional reciprocal lattice of dynaCOF-301a along the (**a**) [100], (**b**) [010], and (**c**) [100] directions, respectively. The obvious diffuse strings along the *c**-axis in these projections indicate disorders in this phase.

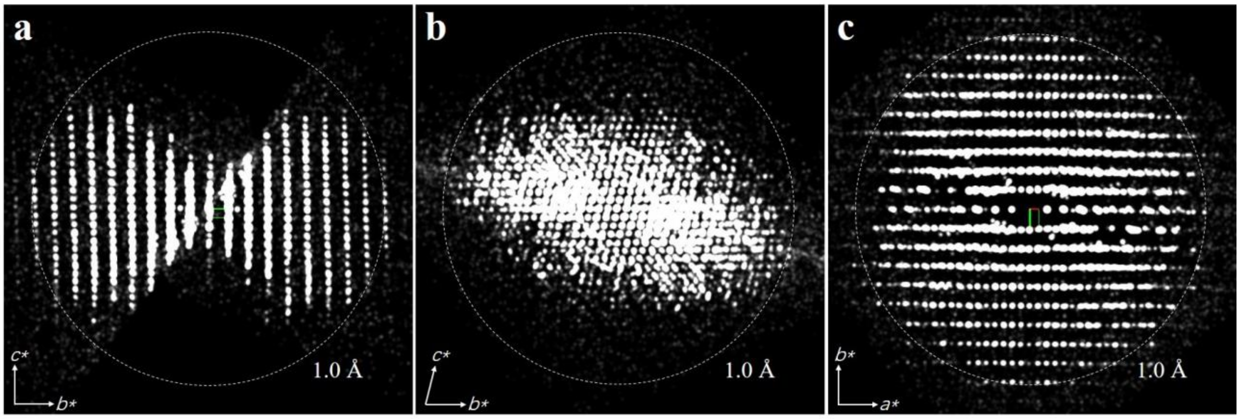

Supplementary Figure 5. Projections of reconstructed three-dimensional reciprocal lattice of dynaCOF-301h along (**a**) [100], (**b**) [010] and (**c**) [001] directions, respectively. The square root of all pixel intensity was used to clearly show the weak diffraction points. The reconstructed reciprocal lattice shows a primitive monoclinic Bravais lattice. And the resolution of the whole dataset is up to 1.0 Å.

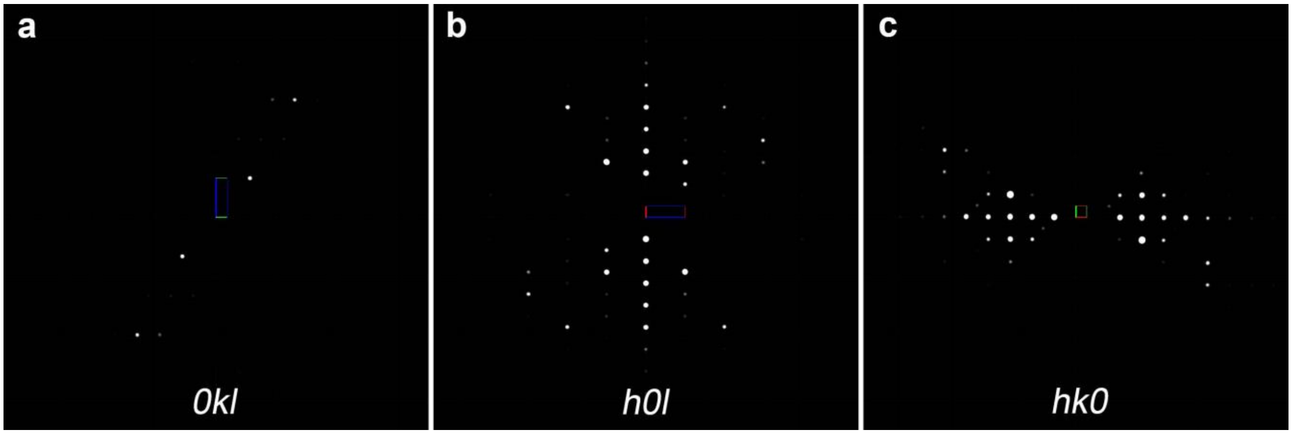

Supplementary Figure 6. The slices cut from the reconstructed three-dimensional reciprocal lattice of 3D ED data of dynaCOF-301s. (a) (0*kl*), (b) (*h*0*l*) and (c) (*hk*0).

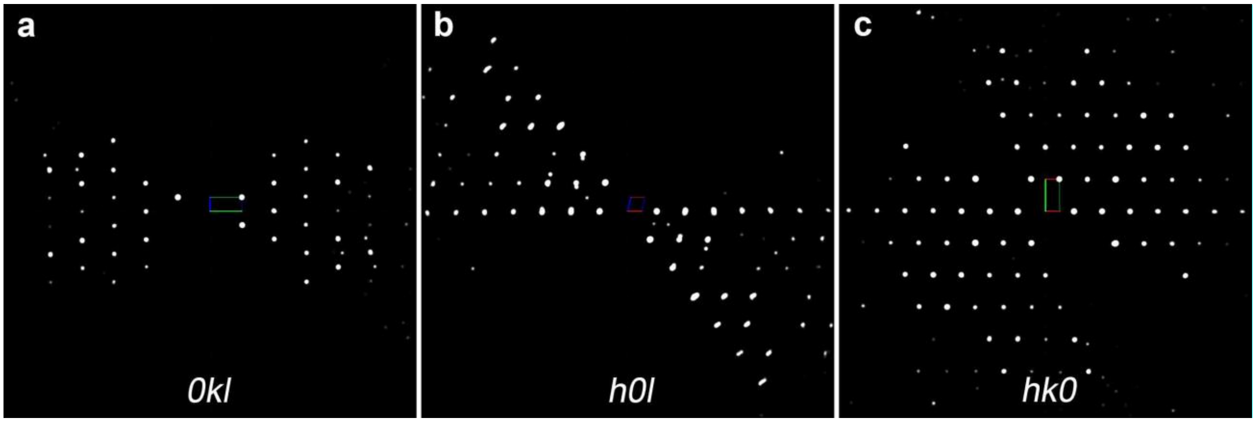

Supplementary Figure 7. The slices cut from the reconstructed three-dimensional reciprocal lattice of 3D ED data of dynaCOF-301h. (a) (0*kl*), (b) (*h*0*l*) and (c) (*hk*0).

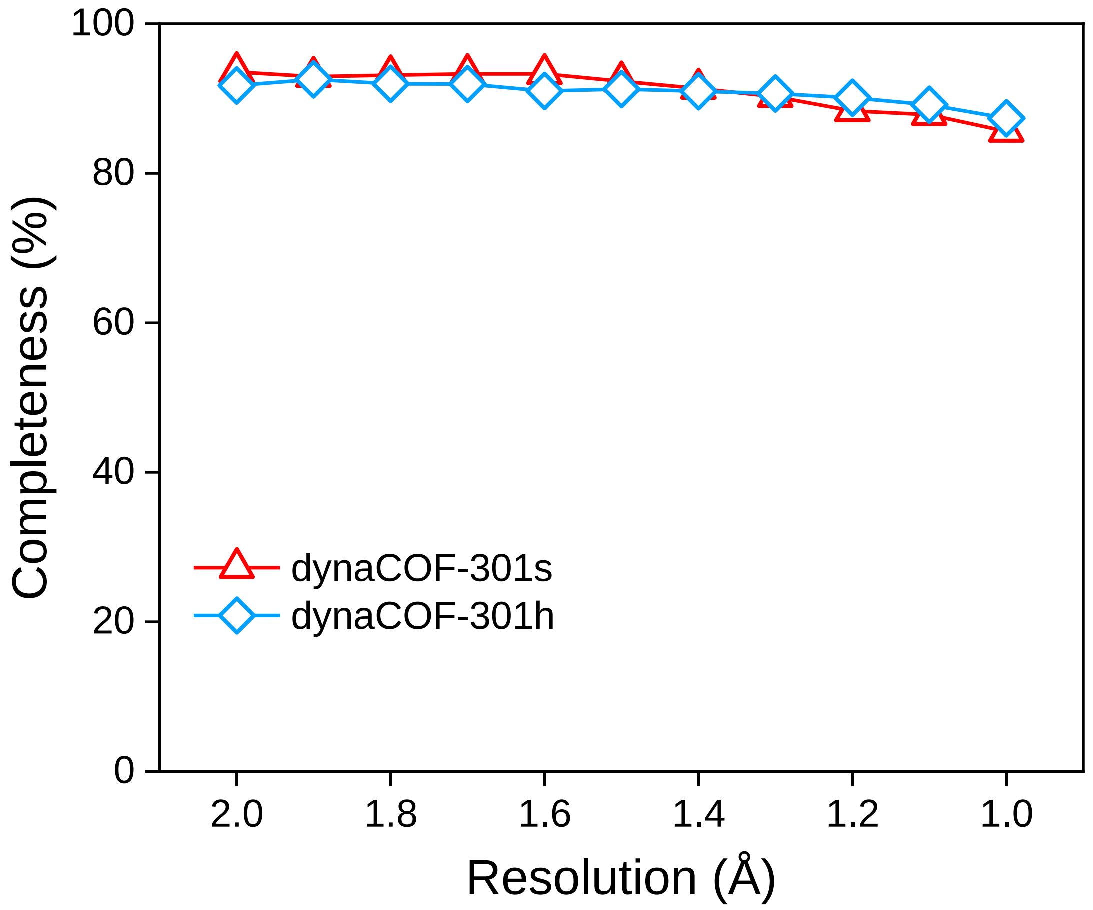

Supplementary Figure 8. The completeness of the 3D ED dataset of dynaCOF-301s and dynaCOF-301h against resolutions.

Supplementary Table 1. ED structure solution and refinement statistic of dynaCOF-301s and dynaCOF-301h.

	**dynaCOF-301s**	**dynaCOF-301h**
Radiation source	200 kV Electron	200 kV Electron
λ (Å)	0.02508	0.02508
Collection mode	Continuous tilting	Continuous tilting
Tilt range(°)	90.2	~100
Crystal size(μm)	1 × 1 × 3	1 × 1 × 3
Formula	$C_{41}H_{28}N_4O_4$	$C_{41}H_{30}N_4O_5$
Z	4	4
Space group	$I4_1/a$ (no. 88)	$I2/a$ (no.15)
a (Å)	27.75	20.70
b (Å)	27.75	8.81
c (Å)	7.5	20.25
α (°)	90	90
β (°)	90	100.52
γ (°)	90	90
V (Å^3)	5276.08	3520.35
Resolution (Å)	1.0	1.0
R_{int} (%)	25.1	43.1
Completeness (%)	85.6	97.4
Total reflections	6355	30311
Unique reflections	1209	3665
$^aR_1$(%)	14.71	24.63
$^bwR_2$(%)	38.40	58.64

$^aR_1 = \Sigma||F_o| - |F_c||/\Sigma|F_o|$; $^bwR_2 = [\Sigma w(F_o^2 - F_c^2)^2/\Sigma w(F_o^2)2]^{1/2}$

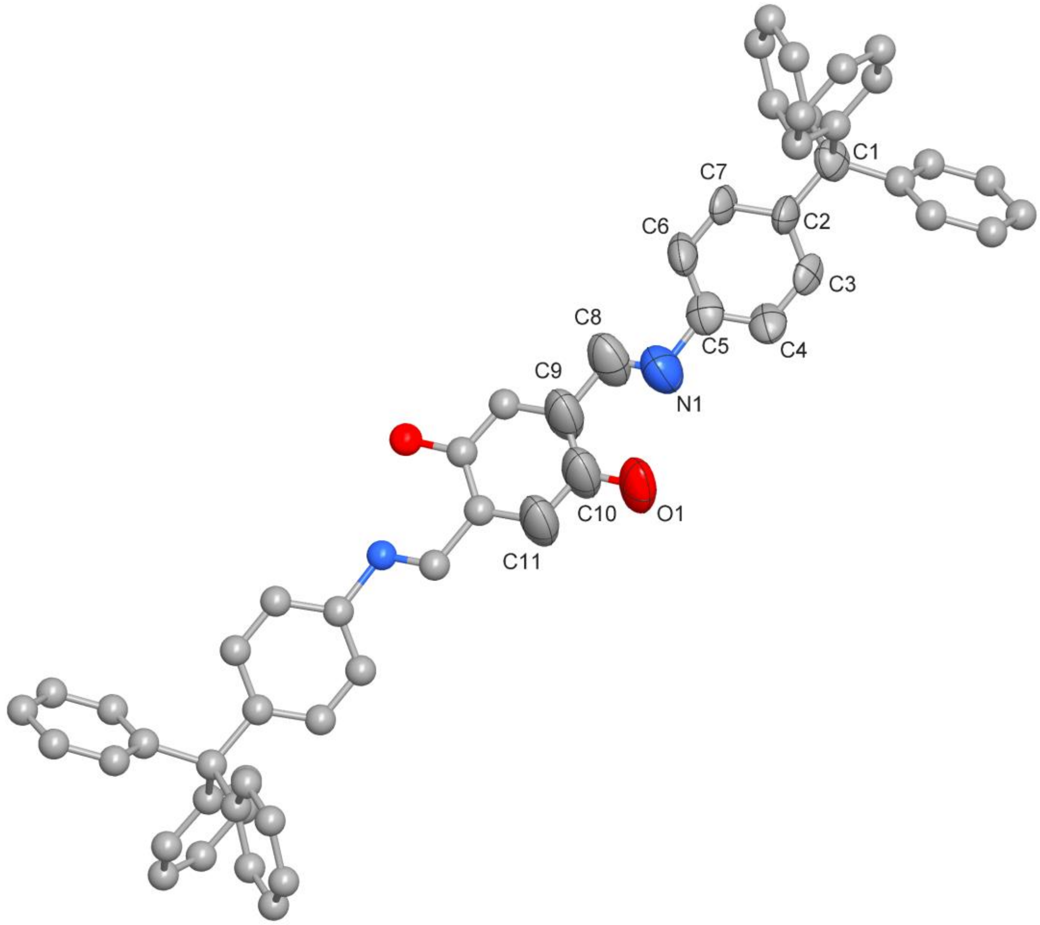

Supplementary Figure 9. ORTEP[1] drawing of the asymmetric unit of dynaCOF-301s (CCDC 2238807). Thermal ellipsoids are displayed with a 50% probability. The grey, blue, and red colour represent C, N and O atoms, respectively. Hydrogen atoms are omitted for clarity; symmetry-related atoms are not labelled and are represented as spheres.

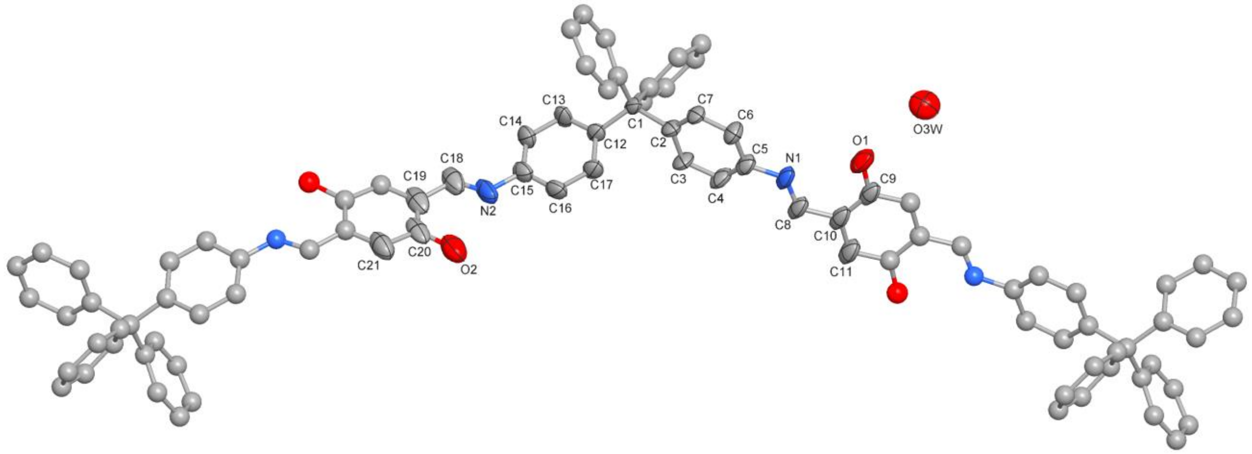

Supplementary Figure 10. ORTEP[1] drawing of the asymmetric unit of dynaCOF-301h (CCDC 2238808). Thermal ellipsoids are displayed with 50% probability, for O3w with 30% probability. The grey, blue, and red colour represent C, N and O atoms, respectively. Hydrogen atoms are omitted for clarity; symmetry-related atoms are not labelled and are represented as spheres.

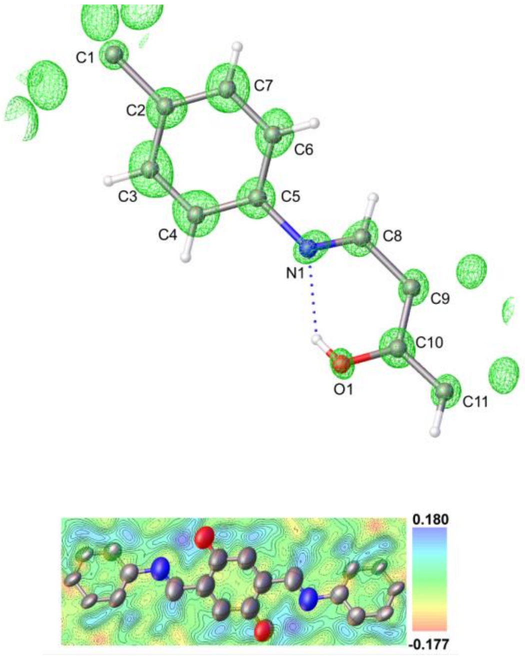

Supplementary Figure 11. The electrostatic potential map of dynaCOF-301s. The grey, blue, and red colour represent C, N and O atoms, respectively.

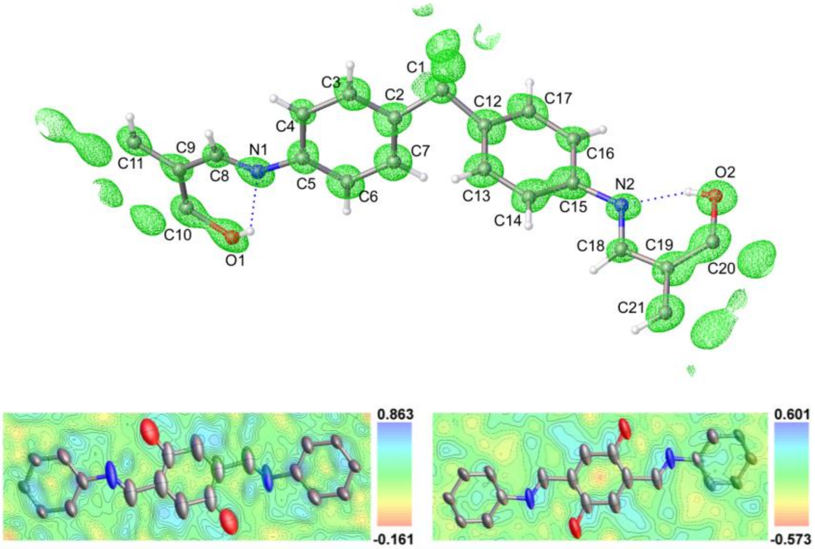

Supplementary Figure 12. The electrostatic potential map of dynaCOF-301h. The grey, blue, and red colour represent C, N and O atoms, respectively.

Supplementary Section 3. Powder X-ray diffraction analyses

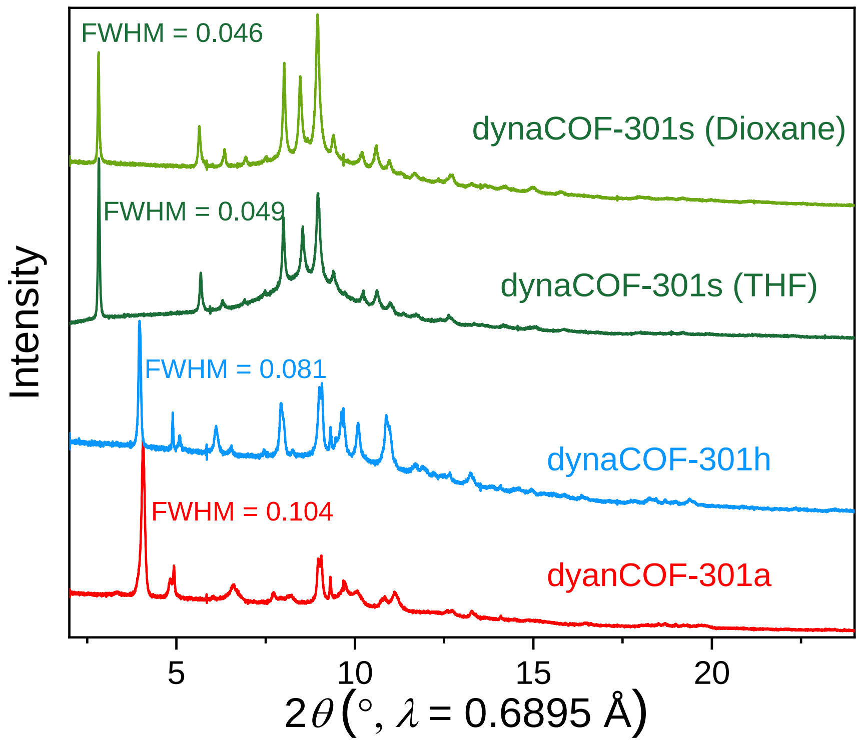

Supplementary Figure 13. Synchrotron PXRD patterns of dynaCOF-301a (red), dynaCOF-301h (blue), and dynaCOF-301s (THF, dark green). dynaCOF-301s (1,4-dioxane, light green).

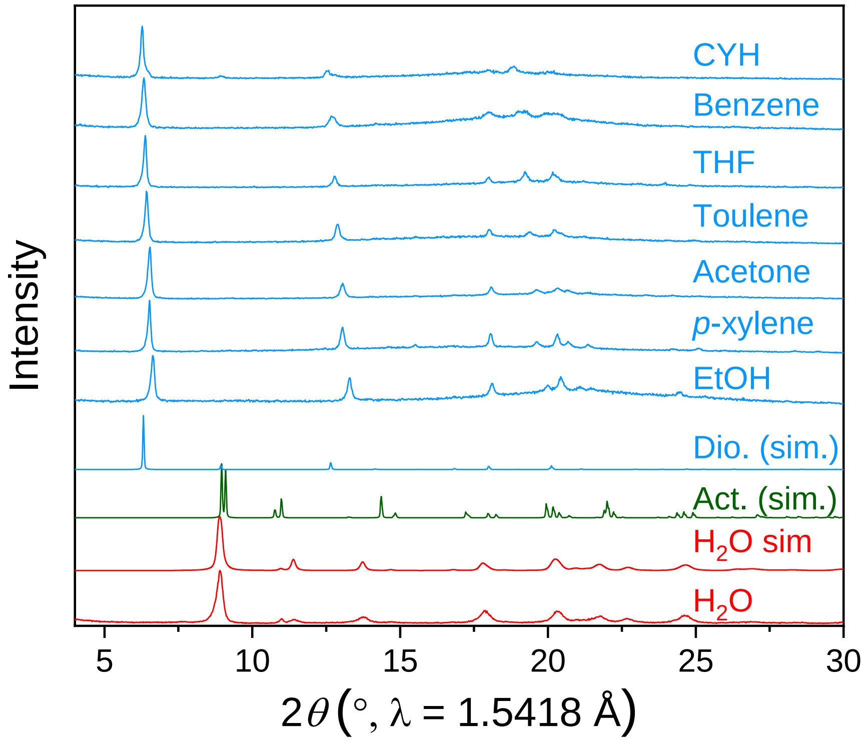

Supplementary Figure 14. PXRD patterns of dynaCOF-301 upon inclusions of organic solvents and water.

Supplementary Table 2. Fractional atomic coordinates for dynaCOF-301s (THF) (Rietveld).

dynaCOF-301s (THF)					
Space group: *I*4_1*/a* (no.88); *a* = *b* = 27.68687 Å; *c* = 7.26611 Å; *α* = *β* = *γ* = 90˚; *V* = 5569.929 Å^3; Z =4					
atom label	*x*	*y*	*z*	B_{iso}	Occupancy
C1	0.00000	0.25000	0.12500	5.00000	1.00
C2	-0.00222	0.29555	0.24867	1.14854	1.00
C3	-0.04291	0.32649	0.24583	1.14854	1.00
C4	-0.04438	0.36716	0.36140	1.14854	1.00
C5	-0.00736	0.37484	0.48886	1.14854	1.00
C6	0.0323	0.34411	0.49505	1.14854	1.00
C7	0.0337	0.30308	0.38273	1.14854	1.00
C8	0.02206	0.42754	0.74790	1.00000	1.00
C9	0.00838	0.46216	0.87740	1.00000	1.00
C10	-0.01198	0.50537	0.81665	1.00000	1.00
C11	-0.02697	0.54004	0.94494	1.00000	1.00
C12s	0.3488745	0.5745712	0.9397677	1.00000	1.00
C13s	0.306797	0.5491292	0.8505376	1.00000	1.00
C14s	0.2946462	0.5846421	0.6985171	1.00000	1.00
C15s	0.344299	0.6027888	0.6414846	1.00000	1.00
N1	-0.00844	0.41705	0.60878	1.00000	1.00
O1	-0.01639	0.51404	0.63377	1.14854	1.00
O2s	0.3758869	0.5939219	0.7920713	1.00000	1.00

Supplementary Table 3. Fractional atomic coordinates for dynaCOF-301a (Rietveld).

**dynaCOF-301a**<br>Space group: *I*2/*a* (no.15); a = 20.51356 Å, b = 8.84134 Å; c = 20.39424 Å; $\alpha = \gamma$ =90˚; β = 107.14376˚; V = 3534 Å^3; Z = 4					
atom label	*x*	*y*	*z*	B_{iso}	Occupancy
C1	0.76341	0.51124	0.061	1.00000	1.00
C2	0.70746	0.43127	0.07041	1.00004	1.00
C3	0.7168	0.30743	0.11369	1.00004	1.00
C4	0.78251	0.25729	0.1482	1.00004	1.00
C5	0.83872	0.33579	0.14033	1.00004	1.00
C6	0.82955	0.46242	0.09745	1.00004	1.00
C7	0.75133	0.06493	0.21662	1.00004	1.00
C8	0.75452	0.90629	0.23904	1.00004	1.00
C9	0.81254	0.81294	0.24821	1.00004	1.00
C10	0.80661	0.65834	0.25692	1.00004	1.00
C11	0.69063	0.72915	0.99734	1.00004	1.00
C12	0.67675	0.77224	0.05837	1.00004	1.00
C13	0.63525	0.89505	0.05968	1.00004	1.00
C14	0.60761	0.98098	1.00137	1.00004	1.00
C15	0.61934	0.93944	0.93958	1.00004	1.00
C16	0.66043	0.81431	0.93761	1.00004	1.00
C17	0.53452	0.20195	0.96426	1.00004	1.00
C18	0.51287	0.35009	0.98375	1.00004	1.00
C19	0.5172	0.39072	0.05226	1.00004	1.00
C20	0.50603	0.53975	0.06752	1.00004	1.00
C21	0.76341	0.51124	0.06100	1.00004	1.00
N1	0.79267	0.11740	0.18484	3.82029	1.00
N2	0.57547	0.11902	1.01033	3.82029	1.00
O1	0.87752	0.86738	0.25276	1.00000	1.00
O2	0.52943	0.28830	0.10647	1.00000	1.00

Supplementary Table 4. Fractional atomic coordinates for dynaCOF-301h (Rietveld).

dynaCOF-301h					
Space group: *I*2/*a* (no.15); a = 20.07474 Å, b = 8.81169 Å; c = 20.25318 Å; $\alpha = \gamma$ =90°; β = 100.51586°; V = 3522.461 Å^3; Z = 4					
atom label	*x*	*y*	*z*	B_{iso}	Occupancy
C1	0.75000	0.61539	0.00000	1.00000	1.00
C2	0.75552	0.51280	0.06302	1.00004	1.00
C3	0.81757	0.44906	0.09572	1.00004	1.00
C4	0.81823	0.32271	0.13866	1.00004	1.00
C5	0.75625	0.26275	0.15092	1.00004	1.00
C6	0.69621	0.32763	0.11933	1.00004	1.00
C7	0.69616	0.44929	0.07577	1.00004	1.00
C8	0.69386	0.03149	0.19188	1.00004	1.00
C9	0.72297	0.88639	0.22413	1.00004	1.00
C10	0.79480	0.86591	0.23542	1.00004	1.00
C11	0.82189	0.72744	0.26019	1.00004	1.00
C12	0.68750	0.71815	0.99385	1.00004	1.00
C13	0.66049	0.78536	0.93214	1.00004	1.00
C14	0.61431	0.90431	0.92906	1.00004	1.00
C15	0.60085	0.96771	0.98810	1.00004	1.00
C16	0.62873	0.90360	0.04954	1.00004	1.00
C17	0.67646	0.78845	0.05194	1.00004	1.00
C18	0.54507	0.22836	0.94454	1.00004	1.00
C19	0.52052	0.36811	0.97440	1.00004	1.00
C20	0.52328	0.38438	0.04540	1.00004	1.00
C21	0.50459	0.51949	0.07076	1.00004	1.00
N1	0.75213	0.12831	0.19113	3.82029	1.00
N2	0.5575	0.09636	0.98513	3.82029	1.00
O1	0.83767	0.98476	0.23114	1.00000	1.00
O2	0.53699	0.26417	0.08833	1.00000	1.00
Ow1	0.937069	0.659117	0.746164	1.00000	1.00
Ow2	0.462851	0.461125	0.266600	1.00000	1.00

Supplementary Table 5. Rietveld refinement structure statistic of dynaCOF-301s (THF), dynaCOF-301a and dynaCOF-301h

	**dynaCOF-301s (THF)**	**dynaCOF-301a**	**dynaCOF-301h**
Molecular	$C_{164}N_{16}O_{16}H_{112}(C_4H_8O)_{16}$ Z = 4	$C_{164}N_{16}O_{16}H_{112}$ Z = 4	$C_{164}N_{16}O_{16}H_{112}\cdot(H_2O)_{16}$ Z = 4
Radiation source	synchrotron X-ray (0.6895 Å)	synchrotron X-ray (0.6895 Å)	synchrotron X-ray (0.6895 Å)
Space group	$I4_1/a$ (no.88)	$I2/a$ (no.15)	$I2/a$ (no.15)
a (Å)	27.68687	20.51356	20.07474
b (Å)	27.68687	8.84134	8.81169
c (Å)	7.26611	20.39424	20.25318
α (º)	90	90	90
β (º)	90	107.14376	100.51586
γ (º)	90	90	90
V (Å^3)	5569.929	3534.47	3522.461
R_{wp} (%)	3.834	5.035	3.620
R_p (%)	2.896	3.570	2.532

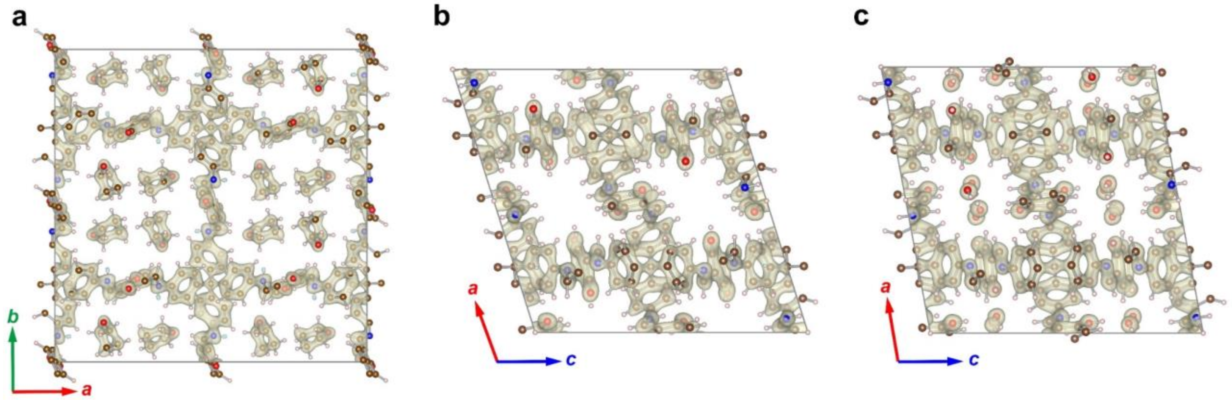

Supplementary Figure 15. Electron density map of **a**) dynaCOF-301s (THF), **b**) dynaCOF-301a and **c**) dynaCOF-301h. These maps were plotted against intensity extracted from synchrotron PXRD pattern after Rietveld refinement. The brown, blue and red colours represent C, N and O atoms.

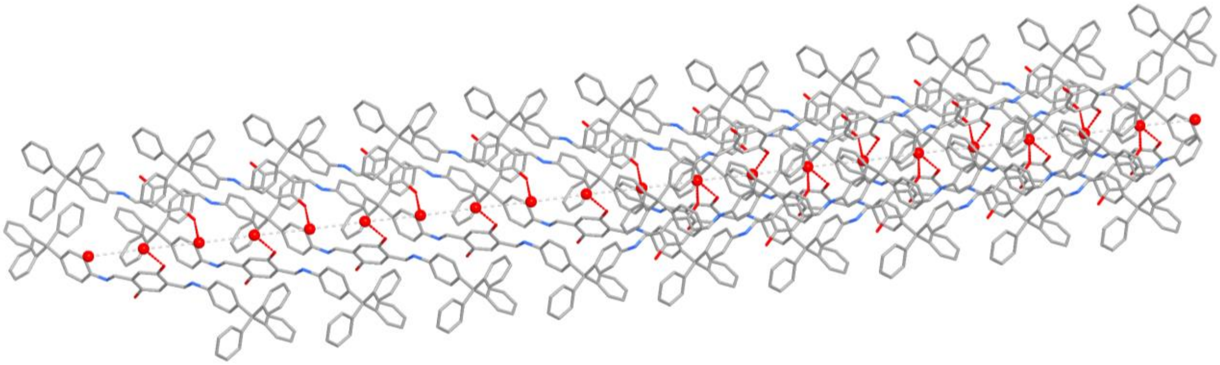

Supplementary Figure 16. The crystal structure of dynaCOF-301h was determined through 3D ED analyses. The weak interactions were found from the water to the nitrogen atom on the wall.

Supplementary Section 4. Gas adsorption and *in-situ* PXRD analyses

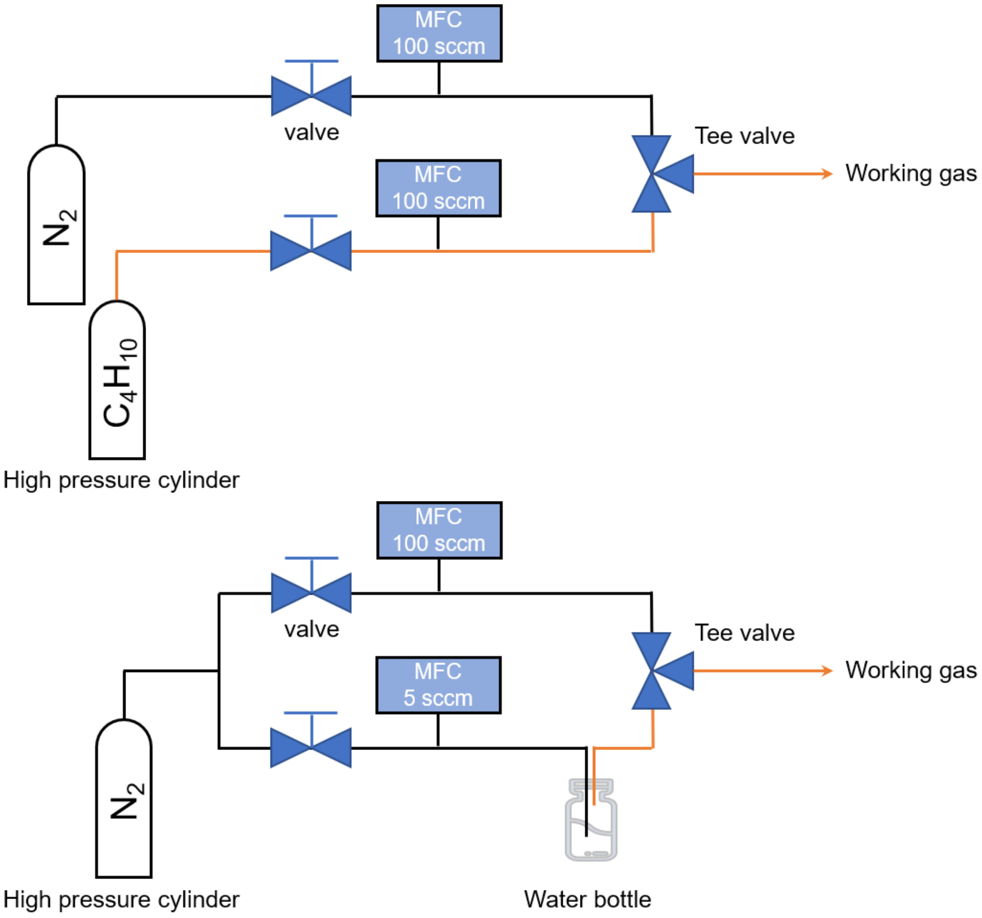

Supplementary Figure 17. Schematic illustration of the customised relative humidity and gas partial pressure controller connected to the *in-situ* PXRD chamber.

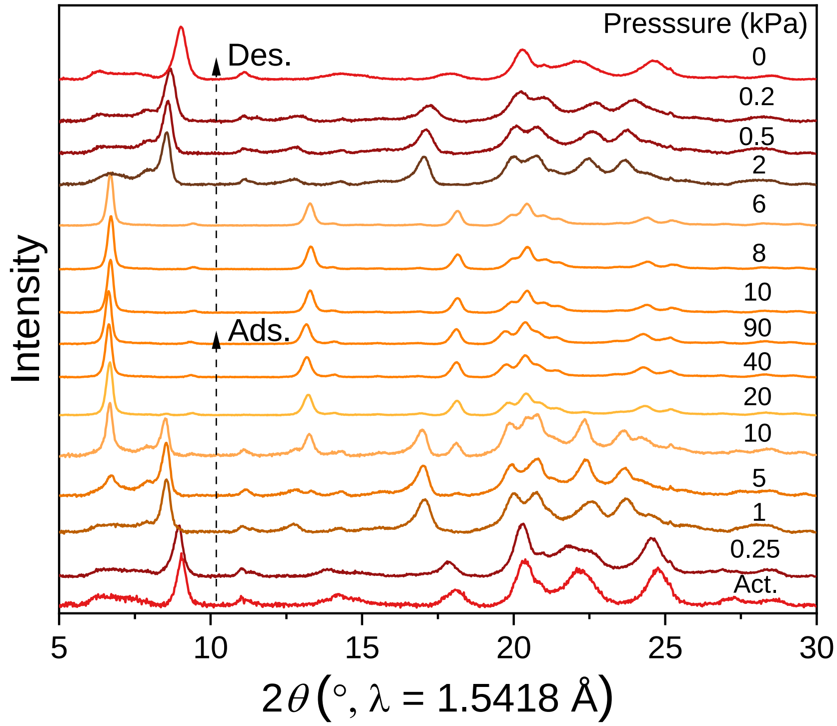

Supplementary Figure 18. *In-situ* PXRD patterns during n-Butane adsorption and desorption of dynaCOF-301 achieved an equilibrium state in different n-Butane pressure at 303 K, showing the reversibility of structural transition upon guest inclusion and removal.

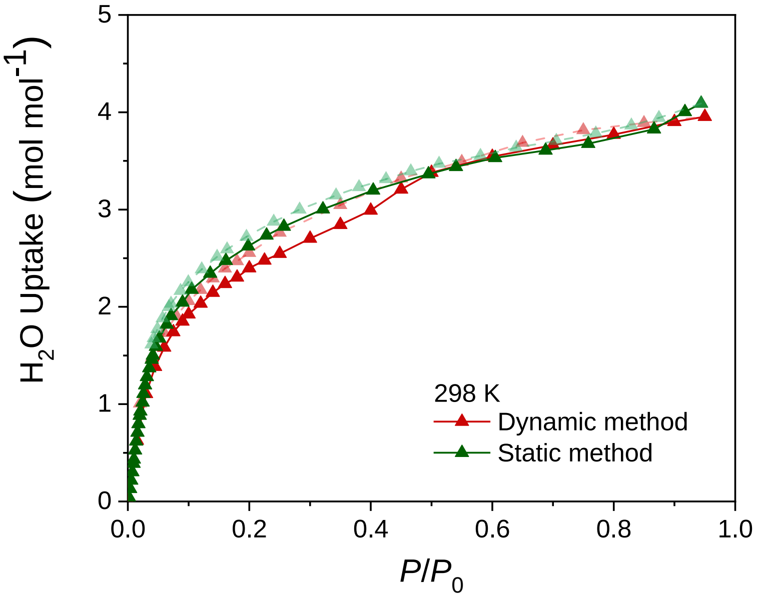

Supplementary Figure 19. The H_2O adsorption isotherms at 298 K (solid line: adsorption, dashed line: desorption) of dynaCOF-301 via DVS and static vapour sorption measurements show a negligible difference.

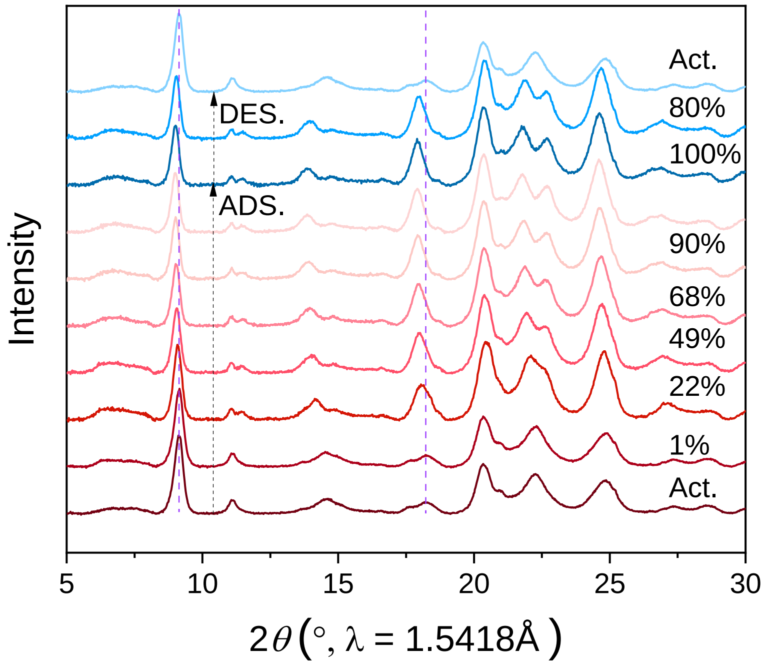

Supplementary Figure 20. *In-situ* PXRD patterns during water adsorption of dynaCOF-301 achieved an equilibrium state in different humidity at 301 K, showing the reversibility of structural transition upon guest filling and removal.

Supplementary Table 6. In-situ PXRD patterns FWHM of peak approximately in 9° of dynaCOF-301 achieved equilibrium state in different humidity, showing significant enhancement on crystallinity.

**Humidity**	**0**	**1 %**	**22 %**	**49 %**	**68%**	**90%**	**100%**
Peak position （°）	9.14	9.10	9.08	9.03	9.00	8.99	8.98
FWHM	0.40	0.40	0.37	0.35	0.35	0.34	0.34

Supplementary Section 5. Diffuse reflectance spectroscopy

Supplementary Table 7. Energy bands of activated and solvent-dosed dynaCOF-301 samples.

Solvent	N/A	Dioxane	Hexane	Toluene	MeCN	THF	Water
Band 1	2.19	2.16	2.17	2.19	2.13	2.1	N/A
Band 2	1.83	N/A	N/A	N/A	N/A	N/A	1.83
Colour	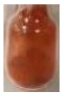	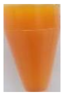	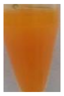	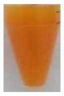	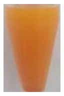	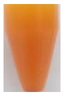	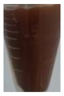

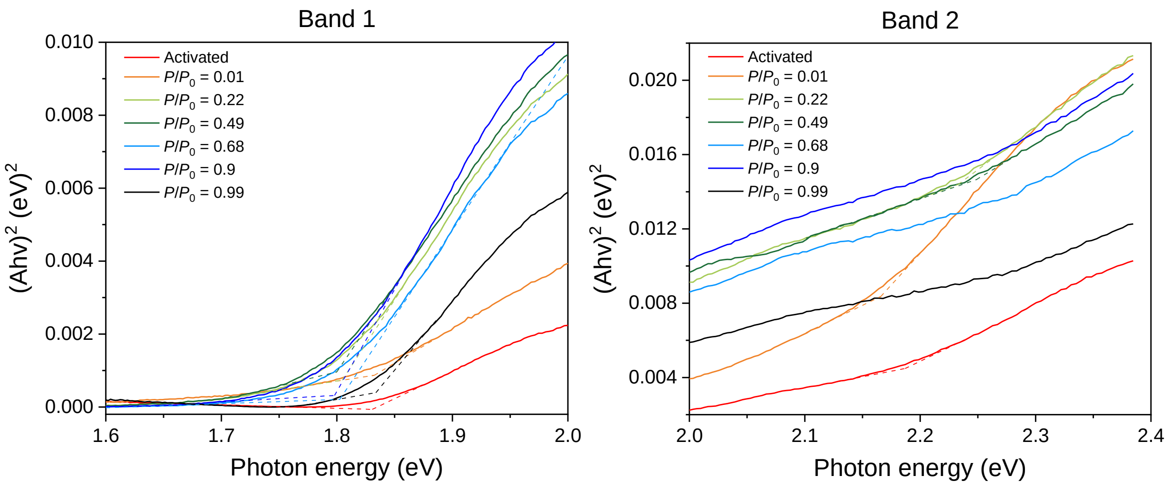

Supplementary Figure 21. The energy band of activated and water-dosed dynaCOF-301 were obtained according to the *Tauc* plot method from DRS.

Supplementary Table 8. Energy bands of activated and water-dosed dynaCOF-301 samples.

P/P_0	0	0.01	0.22	0.49	0.68	0.9	0.99
Band 1	1.83	1.83	1.81	1.80	1.80	1.80	1.83
Band 2	2.19	2.17	2.23	2.20	N/A	N/A	N/A

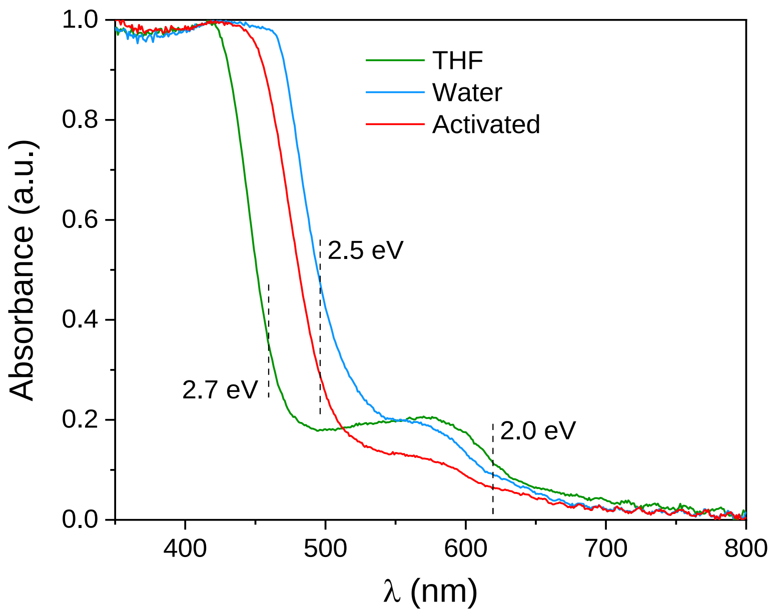

Supplementary Figure 22. Diffuse reflectance spectra (DRS) of activated and solvent-dosed dynaCOF-300.

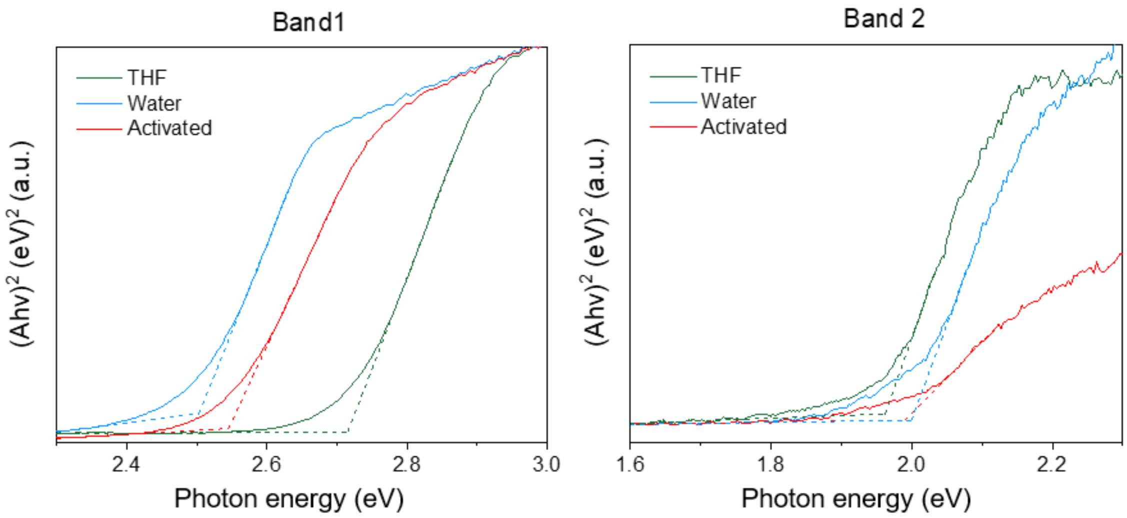

Supplementary Figure 23. Energy bands of activated and solvent-dosed dynaCOF-300 were obtained according to the *Tauc* plot method from DRS.

Supplementary Table 9. Energy band of activated and water-dosed dynaCOF-300 samples.

Solvent	N/A	THF	Water
Band 1	2.55	2.72	2.50
Band 2	1.99	1.96	2.00
Colour	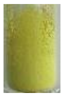	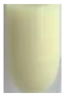	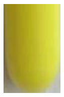

Supplementary Section 6. Solid-state NMR spectroscopy

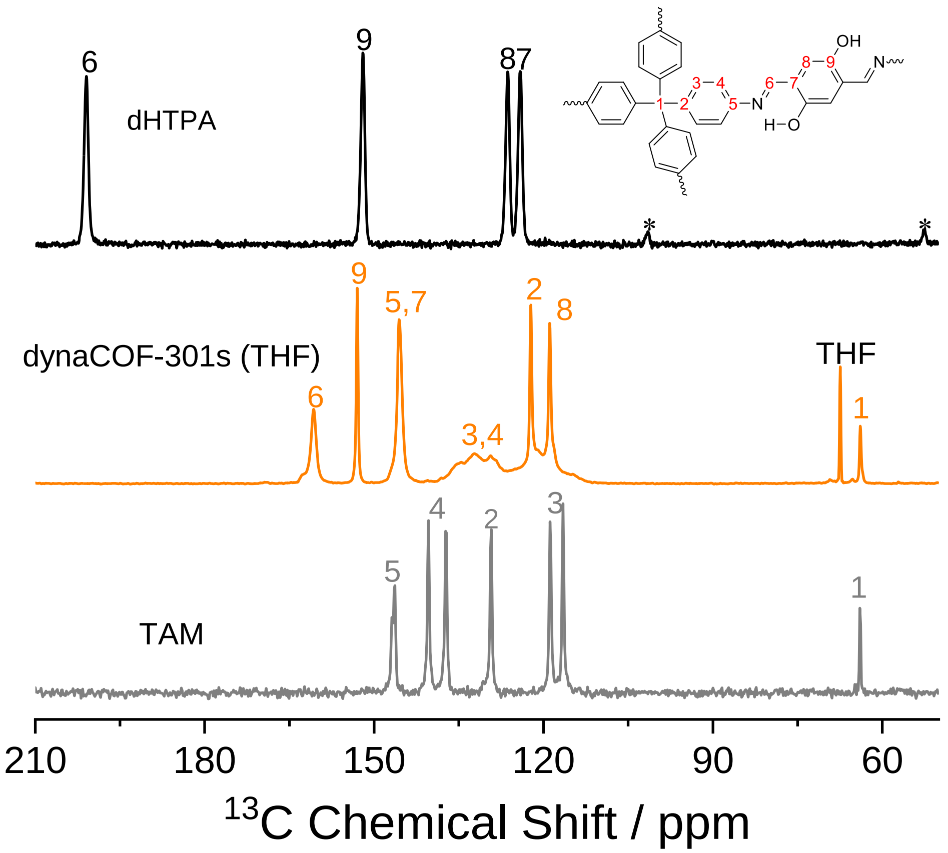

Supplementary Figure 24. ^{13}C SSNMR of dynaCOF-301s (THF) compared with TAM and dHTPA, spinning sidebands are marked by asterisks.

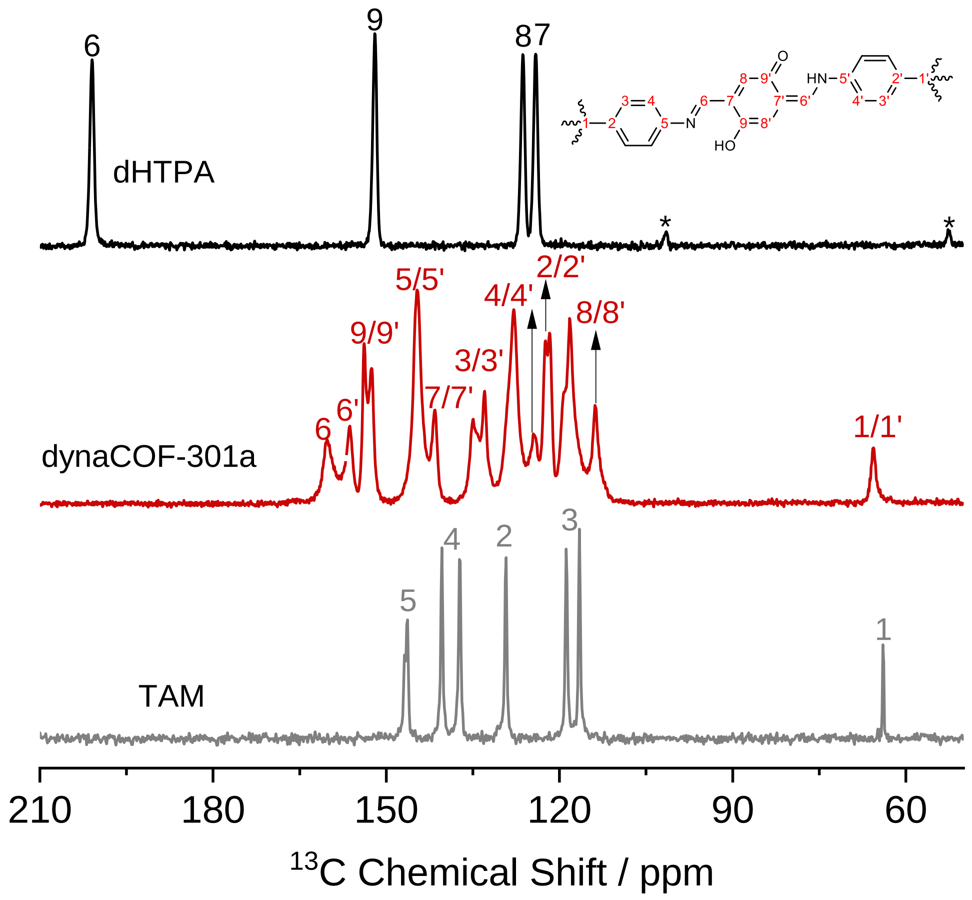

Supplementary Figure 25. ^{13}C SSNMR of dynaCOF-301a compared with TAM and dHTPA, spinning sidebands are marked by asterisks.

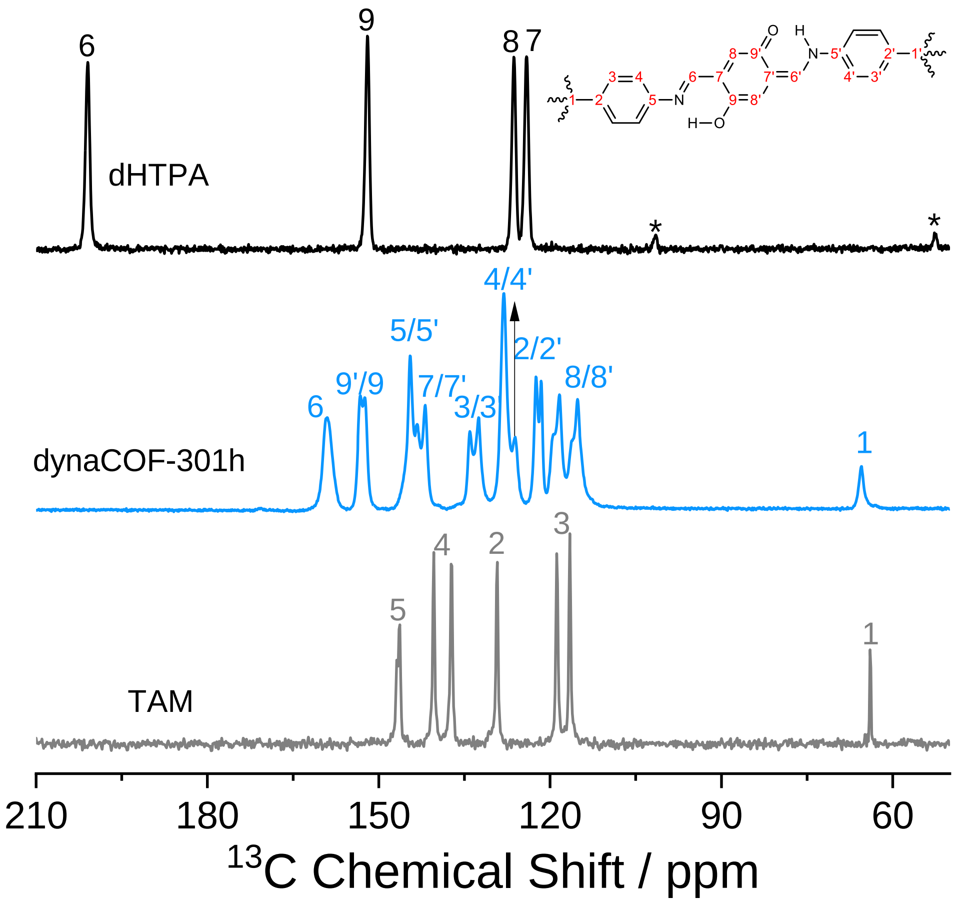

Supplementary Figure 26. ^{13}C SSNMR of dynaCOF-301h compared with TAM and dHTPA, spinning sidebands are marked by asterisks.

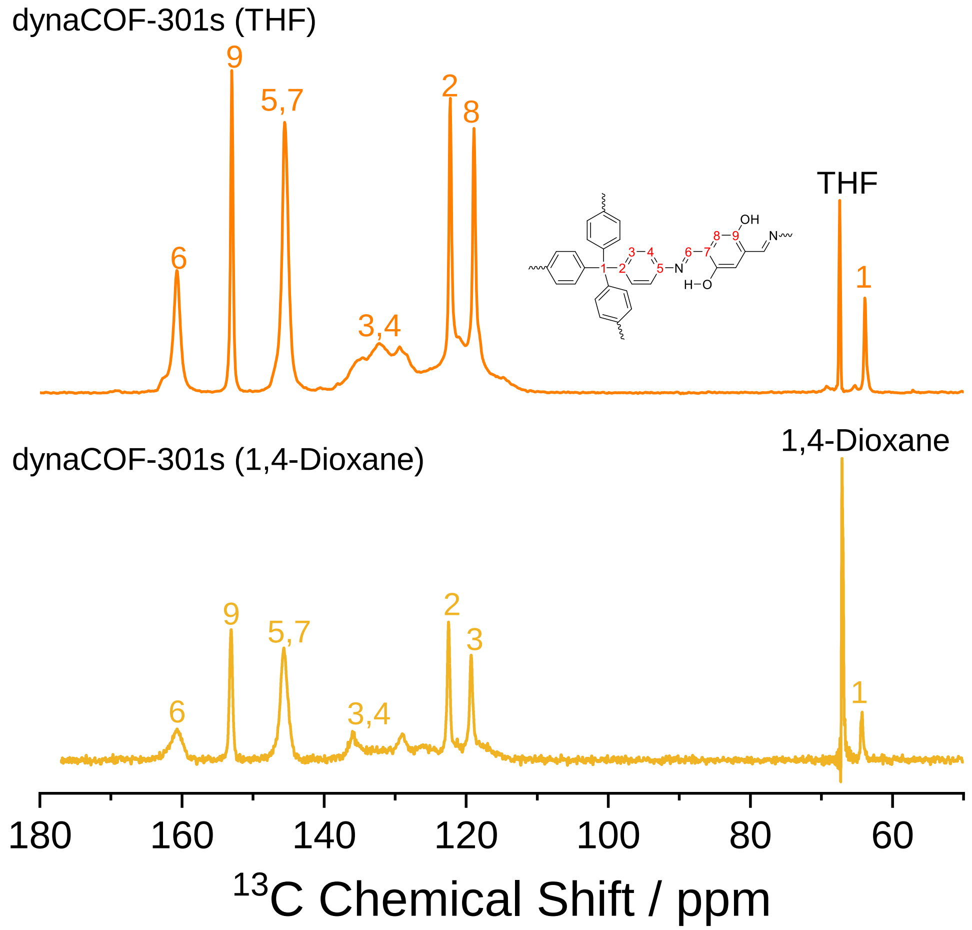

Supplementary Figure 27. ^{13}C SSNMR of dynaCOF-301s (1,4-Dioxane) and dynaCOF-301s (THF).

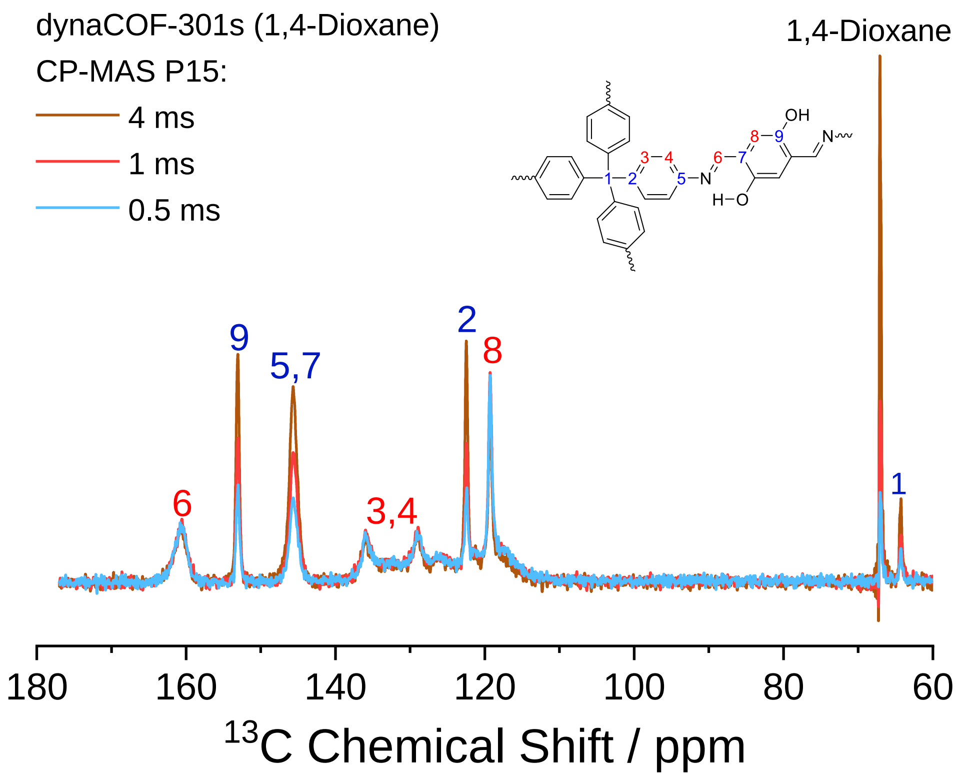

Supplementary Figure 28. ^{13}C SSNMR of dynaCOF-301s (1,4-Dioxane) with different values of contact time to attribute quaternary carbon atoms (carbon number 1,2 5,7,9)

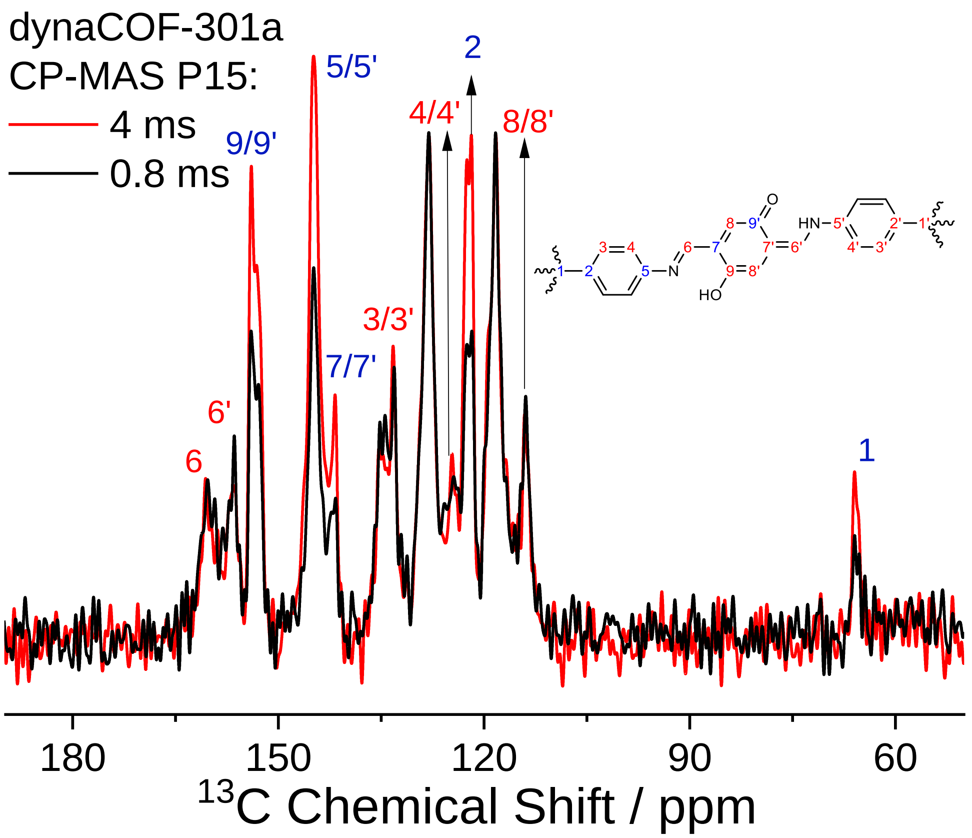

Supplementary Figure 29. ^{13}C SSNMR of dynaCOF-301a with different values of contact time to attribute quaternary carbon atoms (carbon number 1,2 5,7,9)

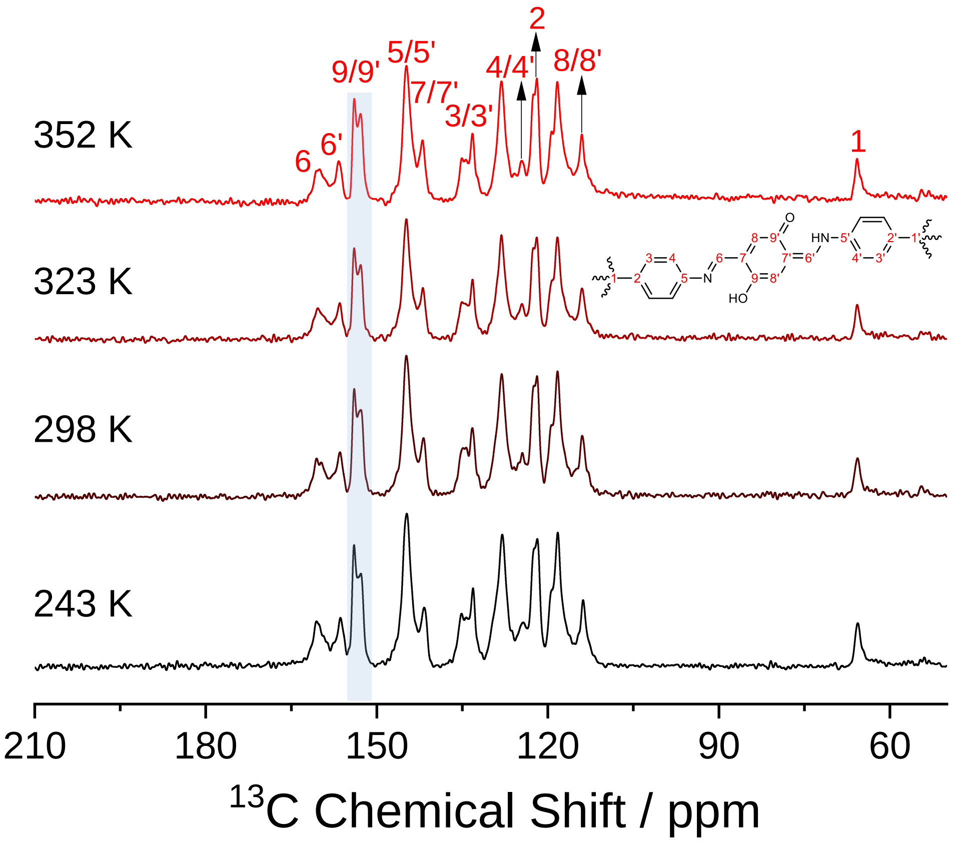

Supplementary Figure 30. ^{13}C SSNMR of dynaCOF-301a in varied temperatures, showing a distinguishable effect on the relative intensity of C9 and C9’, implying temperature-dependent tautomerism of moieties.

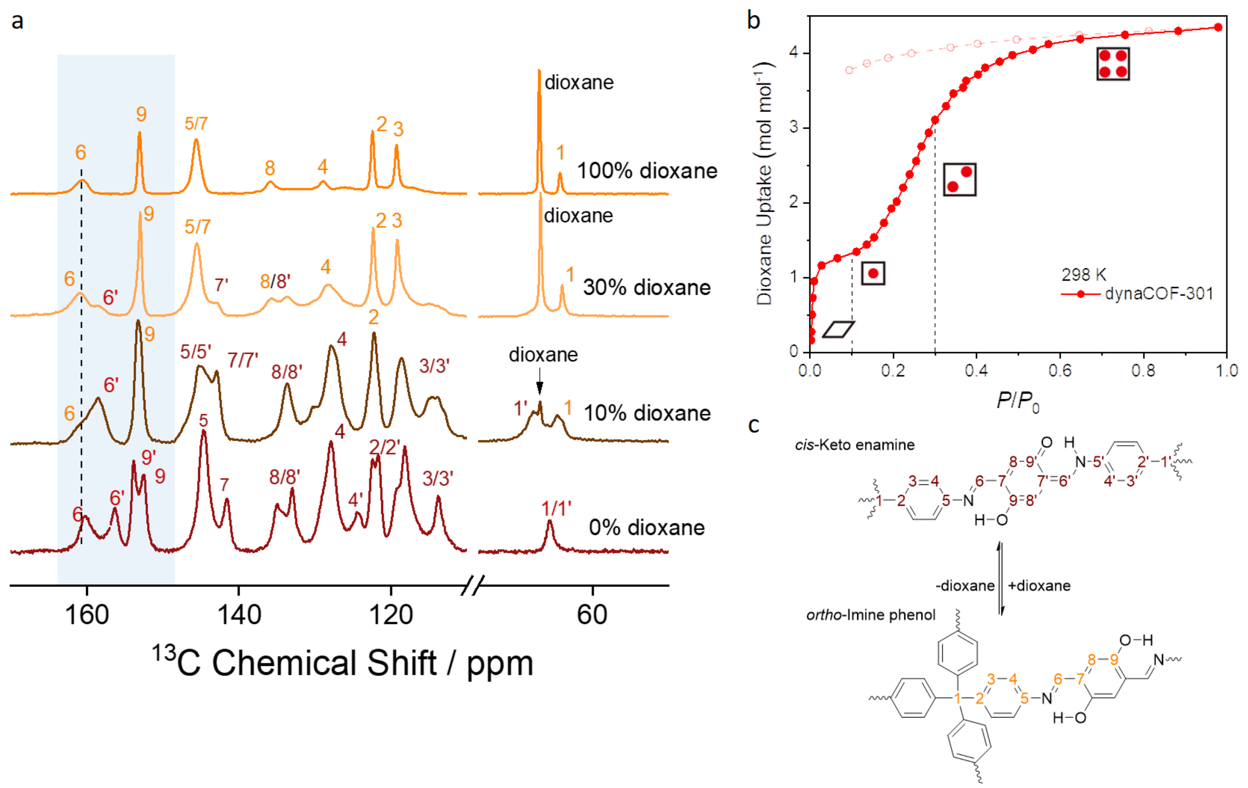

Supplementary Figure 31. *ex-situ* ^{13}C SSNMR of dynaCOF-301 loading with different pressures of dioxane vapour, showing a distinguishable effect on the chemical shift, implying guest-dependent tautomerism of moieties.

Supplementary Section 7. Molecular Dynamics Simulation

To understand the dynamic change process of dynaCOF-301 structure, we constructed the free energy profiles of the system as a function of volume by the method proposed by V. Van Speybroeck *et al.*[2]Molecular dynamics (MD) simulations of the NPT ensemble were performed from the dynaCOF-301 extended phase to obtain 30 snapshots with volumes ranging from 3300 to 5700 Å^3 at 112K and 2000 bar. For each snapshot, a simulation in the (N, V, $\sigma_a = 0$, T) ensemble was performed using the Martyna−Tuckerman−Tobias−Klein (MTTK) barostat[2] which samples cell fluctuations. When the system relaxes its structure under constant volume constraints, the mean value of the pressure tensor trace is the hydrostatic pressure p(V). Because hydrostatic pressure is the negative volume derivative of the free energy, the Helmholtz free energy A(V) can be calculated by integrating according to $\Delta \mathrm{A(V)} = -\int_{V_0}^{V} p(V)dV$

The LAMMPS software package with OPLS2005 force field[3] performed all molecular dynamics simulations. In each simulation, the relaxation time was 0.1 ps of Nosé−Hoover thermostat and a cut-off of 12 Å was used. We successfully obtained the free energy profile in the dynamic change process of the dynaCOF-301 structure through thermodynamic integration.

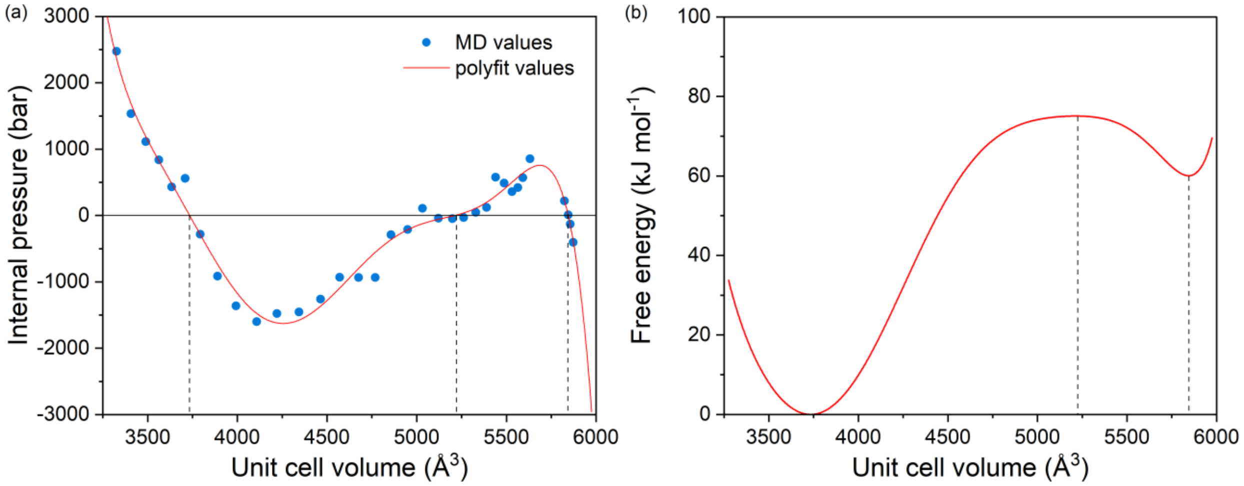

Supplementary Figure 32. Fitted internal pressure P (left) and free energy F (right) profiles as a function of the constrained unit cell volume V for dynaCOF-301, resulting from (N, V, $\sigma_a = 0$, T) simulations at $T = 112$ K.

Supplementary References

[1] Farrugia, L. J. *J. Appl. Cryst.* **45**, 849-854 (2012).

[2] Rogge, S. M. J.; Vanduyfhuys, L.; Ghysels, A.; Waroquier, M.; Verstraelen, T.; Maurin, G.; Van Speybroeck, V. *J Chem Theory Comput.* **11**, 5583-5597 (2015).

[3] Banks, J. L.; Beard, H. S.; Cao, Y.; Cho, A. E.; Damm, W.; Farid, R.; Felts, A. K.; Halgren, T. A.; Mainz, D. T.; Maple, J. R.; Murphy, R.; Philipp, D. M.; Repasky, M. P.; Zhang, L. Y.; Berne, B. J.; Friesner, R. A.; Gallicchio, E.; Levy, R. M. *Journal of Computational Chemistry* **26**, 1752-1780 (2005).
