# Supplementary material for: Symmetry-breaking dynamics in a tautomeric 3D covalent organic framework
Source: Nat Commun. 2023 Jul 14;14:4215. doi: 10.1038/s41467-023-39998-x (PMC10349083; doi:10.1038/s41467-023-39998-x)
Supplement: Supplementary file 3 — Description of Additional Supplementary Files [file 41467_2023_39998_MOESM3_ESM.pdf]

Description of additional supplementary files

Supplementary Movie 1 : Visualization of structural dynamics using molecular simulations.
